# Optimising parent selection in plant breeding: comparing metaheuristic algorithms for genotype building

**DOI:** 10.1007/s00122-025-05028-1

**Published:** 2025-09-06

**Authors:** S. Yadav, S. Dillon, M. McNeil, E. Dinglasan, R. Mago, P. Dodds, L. Hickey, B. J. Hayes

**Affiliations:** 1 Queensland Alliance for Agriculture and Food Innovation, University of Queensland, Brisbane, Australia; 2 CSIRO Agriculture & Food, GPO Box 1700, Canberra, ACT 2601 Australia; 3 CSIRO Agriculture & Food, St Lucia, QLD 4067 Australia

## Abstract

Stacking desirable haplotypes across the genome to develop superior genotypes has been implemented in several crop species. A major challenge in Optimal Haplotype Selection is identifying a set of parents that collectively contain all desirable haplotypes, a complex combinatorial problem with countless possibilities. In this study, we evaluated the performance of metaheuristic search algorithms (MSAs)—genetic algorithm (GA), differential evolution (DE), particle swarm optimisation (PSO), and simulated annealing (SA) for optimising parent selection under two genotype building (GB) objectives: Optimal Haplotype Selection (OHS) and Optimal Population Value (OPV). Using a diverse wheat population of 583 lines genotyped for 29,972 SNPs, forming 7645 haplotype blocks and phenotyped for stripe rust scores, we assessed each algorithm’s performance across fitness optimisation, convergence speed, and computational efficiency. GA consistently achieved high fitness and rapid convergence, while DE showed robustness but required longer runtime and careful tuning. PSO performed well under the OHS criterion but was less effective for OPV. SA, although computationally lighter, was less consistent in finding optimal solutions. Simulation over 100 breeding cycles showed that OHS outperformed both OPV and GEBV-based selection in long-term genetic gain and diversity retention. OHS maintained heterozygosity and additive variance, which are key for sustainable improvement, while GEBV selection led to early allele fixation. Our findings underscore the potential of GB strategies that prioritise the collective performance of parent sets rather than individual ranking to enhance selection outcomes in genomic-assisted breeding programmes.

## Introduction

In plant breeding, the primary objective is to select parent lines that improve key traits such as yield, disease resistance, and tolerance to environmental stresses, which are predominantly governed by quantitative inheritance. Historically, breeders have relied on phenotypic assessments to make these selections. However, advancements in genomic technologies have enabled a shift towards genomic selection (GS), where the breeding potential of individuals is estimated through genomic estimated breeding values (GEBVs) (Heffner et al. 2009; Meuwissen et al. 2001). While GS has accelerated breeding cycles and improved selection accuracy by focusing on immediate genetic gains, it can reduce genetic diversity over time (Daetwyler et al. 2015; Lin et al. 2016; Makanjuola et al. 2020). Selecting only the top individuals based on GEBVs may lead to the loss of rare favourable alleles, potentially compromising long-term breeding objectives and the durability of traits like disease resistance, especially against persistent threats such as rust in wheat.

Unlike GEBV-based selection, which ranks individuals by their estimated breeding value, genotype building (GB) strategies aim to assemble a superior genotype by combining the best chromosome segments across the genome. These strategies allow for selecting individuals who may not rank highly in GEBVs but carry rare or high-value (favourable) segments, enhancing long-term genetic diversity and cumulative gain. To address the limitations of traditional GS and promote long-term genetic improvement, GB strategies such as the Optimal Haplotype Selection (OHS) method (Kemper et al. 2012) and the Optimal Population Value (OPV) (Goiffon et al. 2017) have been proposed. Both selection criteria evaluate parents as a group, calculating a combined fitness value for the set rather than assessing individuals separately. The GB approaches optimise the selection of founder parents by identifying the best possible combinations of chromosome segments across the genome. The aim is to construct “ultimate genotypes” by stacking favourable haplotypes across the genome. These approaches have shown promise in crops such as wheat and chickpea, where genotype constructed from the best haplotype segments outperformed the best current lines by several folds in traits like yield in wheat under varying levels of inputs and in chickpeas for the 100-seed weight (Varshney et al. 2022; Voss-Fels et al. 2019). By strategically selecting groups of individuals likely to produce superior progeny when crossed, GB strategies can, in some cases, enhance long-term breeding outcomes through strategic preservation of genetic diversity and favourable allele combinations (Villiers et al. 2024).

However, implementing GB strategies poses significant computational challenges due to the vast number of potential parent combinations, making the selection problem non-deterministic polynomial-hard (NP-hard). For example, selecting 50 founder parents from a breeding population of 583 lines results in approximately 10^74^ possible combinations. This complexity transforms the task into a combinatorial optimisation problem. Traditional deterministic optimisation algorithms, such as linear and mixed-integer programming, have been widely applied to solve complex decision-making problems in agriculture settings, including crop harvesting scheduling, land allocation, and supply chains (Custodio et al. 2024; Mirzaei et al. 2024). Mixed-integer linear programming has been used to optimise mating plans while maximising expected progeny value (EPV) under mating constraints (Aliloo et al. 2017; Yadav et al. 2023). However, such methods require the marginal contribution of each possible mating pair to be evaluated independently of others, which is unsuitable for GB problems where fitness depends on the collective haplotype composition of the entire group.

**Fig. 1:**
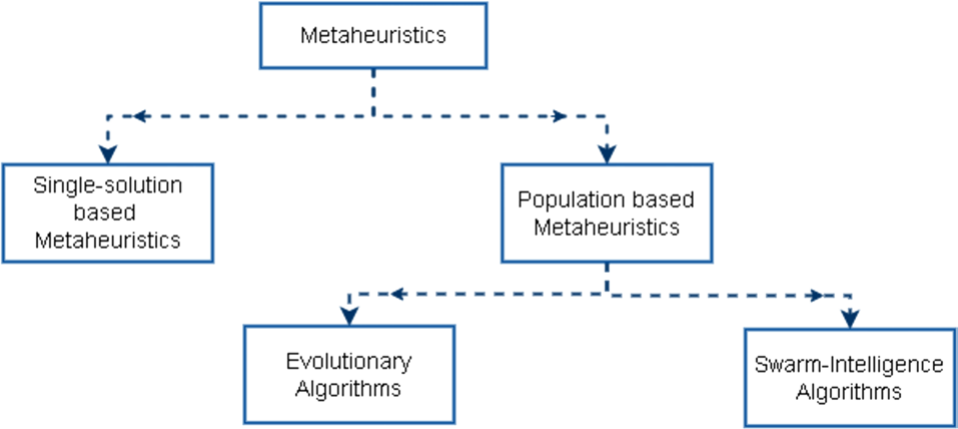
Classification of metaheuristics into single-solution-based and population-based algorithms. Population-based metaheuristics are further categorised into evolutionary algorithms and swarm-intelligence algorithms, which are widely employed for optimisation problems due to their ability to explore and exploit the search space efficiently

Metaheuristic search algorithms (MSAs) offer a promising alternative for tackling such complex optimisation problems due to their flexibility and exploring large, nonlinear, and high-dimensional solution spaces (Fig. 1) (Rajwar et al. 2023). Inspired by natural processes like evolution and swarm behaviour, MSAs balance exploration (diversifying the search) and exploitation (refining potential solutions), increasing the likelihood of identifying global or near-global optima. Unlike traditional methods, MSAs do not require gradient information and are well suited to problems like approaches where fitness cannot be marginalised.

MSAs—such as genetic algorithms (GA) (Bremermann 1958; Holland 1975), differential evolution (DE) (Kinghorn 2011; Storn and Price 1997), particle swarm optimisation (PSO) (Kennedy and Eberhart 1995), and simulated annealing (SA) (Brooks and Morgan 1995; Kirkpatrick et al. 1983), have been successfully applied in agricultural domains. GAs have been used to optimise mating plans and selection schemes (Akdemir & Sánchez, 2016; Kemper et al. 2012; Villiers et al. 2024), while DE has been widely implemented in mate selection problems, including in tools like AlphaMate (Gorjanc & Hickey, 2018). Notable examples include optimising the mating of *Pinus taeda L.* and mate selection in aquaculture breeding under various scenarios using the DE algorithm (Goda and Isik 2024; Yoshida et al. 2017). PSO has been used in optimising agricultural data analysis techniques and supply chain models, while SA has supported breeding decisions by balancing genetic gain and diversity or identifying heterotic groups in wheat (D’Agaro 2012; Meuwissen and Sonesson 2004; Zhao et al. 2015).

Although optimisation algorithms have been applied in various agricultural contexts, there is a lack of comprehensive comparative studies evaluating these algorithms, specifically in the context of founder-parent selection. Additionally, many existing studies lack detailed descriptions of algorithmic implementations, limiting reproducibility. While genomic selection frameworks are well established, less attention has been given to designing robust optimisation frameworks that select breeding individuals based on haplotype-level information. This study compares four widely used MSAs-GA, DE, PSO, and SA, for founder-parent selection under GB schemes. Algorithm performance was evaluated using two GB metrics, OHS and OPV, to enable meaningful comparison across different breeding objectives and recombination frameworks.

These fitness functions span a continuum from practically achievable diversity-preserving strategies to theoretical upper-bound potential. This study evaluates how each MSA performs under these GB formulations and benchmarks them against conventional GEBV-based selection. The objectives of this study are: (i) to compare the performance of GA, DE, PSO, and SA algorithms for optimising founder-parent selection within the GB framework; (ii) provide detailed documentation of algorithm implementation and parameterisation to ensure reproducibility; and (iii) assess algorithmic efficiency, runtime, and achieved fitness across GB formulations. Additionally, a simulation study compared the immediate and long-term impacts of GB vs. GEBV-based founder selection.

Using the GB framework, we hypothesise that founder-parent selection using MSAs will outperform conventional GEBV-based selection by maintaining genetic diversity while achieving substantial genetic gain in rust resistance. This research contributes to developing robust optimisation frameworks that prioritise immediate and sustainable breeding outcomes.

## Material and methods

### Theoretical optimisation framework

Optimisation involves finding the best solution from a defined set of alternatives based on an objective function. Formally, the problem of maximising (or minimising) a scalar-valued objective function $f :S\to {\mathbb{R}}$ can be expressed as finding the optimal set:

1 $${\Theta }^{*}\equiv \text{arg} \,max {f\left(\theta \right)}_{\theta \in\Theta }=\left\{{\theta }^{*}\in\Theta :f\left({\theta }^{*}\right)\ge f\left(\theta \right), \forall \theta \in\Theta \right\}$$

where $\Theta \subseteq S$. The set $S\subseteq {\mathbb{R}}^{p}$ defines the search space, i.e. the domain of parameters $\theta =\left({\theta }_{1},\dots .,{\theta }_{p}\right)$, with each ${\theta }_{i}$ bounded by corresponding upper and lower limits. The set $\Theta$ is the feasible search space defined by a set of *p* constraints. The goal is to identify a solution within the search space $S$ that optimises the objective function. The solution set ${\theta }^{*}$(Eq. 1) could be a unique point or a countable set of points. In many practical scenarios, including breeding programme design, the search space is discrete or combinatorial, meaning parameters are selected from a finite or countable set of options.

A general constrained optimisation problem can be formulated as follows:

$${max}_{x}f\left(x\right)$$

$$subject\,to:$$

$$\left\{\begin{array}{c}{g}_{i}\left(x\right)\le 0, i=1, \dots ., m \\ {g}_{i}\left(x\right)=0, i=m+1, \dots ., n \\ {l}_{j}\le {x}_{j}\le {u}_{j}, j=1, \dots ., q\end{array}\right.$$

Here, ${g}_{i}\left(x\right)$ represents the *i*^th^ constraint function and ${x}_{j}$ represents decision variables within defined bounds. Maximisation can be converted into minimisation by setting max $f\left(x\right)=-\text{min}[-f\left(x\right)]$. Similarly, the inequality constraints ${g}_{i}\left(x\right)\le 0$ can be reformulated as -${g}_{i}\left(x\right)\ge 0$, and boundary constraints for the parameters can also be adjusted accordingly. While constraints help restrict the search space, each potential solution must satisfy all of them to be considered valid. This formulation traditionally applies to continuous domains, and it serves as a conceptual scaffold for more complex, discrete, or structurally constrained optimisation problems encountered in genomics.

### Genotype Building (GB) metrics

*Optimal Haplotype Selection* (Kemper et al. 2012) aims to balance short- and long-term genetic gain by selecting individuals that collectively contribute the highest-value chromosome segments. The objective is to ensure that, across the selected population, each genomic locus is represented by two distinct, high-value haplotypes. This constraint avoids chromosome segment-level homozygosity, helping to preserve rare favourable alleles and maintain genetic diversity over successive breeding cycles.

Mathematical Formulation.

Objective:

$$\text{max}\sum_{j=1}^{B}\sum_{i=1}^{S}{a}_{ij}. {x}_{ij}$$

Subject to:

$$\sum_{i=1}^{S}{a}_{ij}=2 for \,all\, j \epsilon \left\{1,\dots \dots ,B\right\}$$

where B is the number of chromosome segments (haplotype blocks), S ⊆ {1,2,…, N} represents the number of candidate individuals (e.g. 50 in this study), *x*_*ij*_ is the estimated value (e.g. haplotype effects) of individual *i* at segment *j, and a*_*ij*_ $\epsilon$ {0,1} is a binary indicator variable, 1 if segment *j* from individual *i* is selected, 0 otherwise.

This formulation ensures that exactly two distinct high-value segments are selected per locus (*j*), prioritising individuals with good haplotypes even if their overall GEBV is not maximal.

*Optimal Population Value* (Goiffon et al. 2017) focuses on estimating the theoretical upper bound of genetic gain by identifying a subset of parents whose combined haplotypes allow the construction of the most favourable hypothetical progeny under idealised recombination scenarios. This method assumes unrestricted recombination between segments, thereby quantifying the maximum genetic potential achievable from the current genetic pool.

Objective:

$$M \times {max}_{n\in S}{ max}_{m\in \{1,\dots ,M\}}\sum_{b=1}^{B}\sum_{l\in H(b)}{A}_{l,m,n} . {e}_{l}$$

where S is the selected subset of individuals, B is the number of haplotype blocks, H(b) is the set of markers in block b, and *A*_*l,m,n*_ $\epsilon$ {0,1} indicates whether individual n $\epsilon$ S carries the major allele at locus *l* on haplotype copy *m*, *e*_*l*_ is the effect of the major allele at locus *l*, M represents number of haplotype copies (2 haplotypes per locus, diploid assumption), and N is the total number of individuals.

### Metaheuristic algorithms

#### Genetic algorithm (GA)

GA, a search method based on Darwin’s evolutionary theory, operates on a population of candidate solutions (Table 1). In GA, a population of candidate solutions (called “*individuals*”) is represented as binary selection vectors, where each vector is known as a *chromosome*, and each bit indicates inclusion (“1”) or exclusion (“0”) of an item. The algorithm evolves this population across generations through selection, crossover, and mutation, guided by a fitness function, until a termination criterion, such as a maximum number of generations or convergence, is met.

**Table 1:** Comparative analysis of metaheuristic algorithms for optimisation: inspirations, search approaches, strengths, and limitations

Algorithm	Inspiration	Search strategy	Strengths	Limitation
GA	Natural selection	Population-based	Good for diverse, complex search spaces	Can be slow for large datasets
DE	Evolutionary process	Population-based	Simple, efficient, robust	May require careful parameter tuning
PSO	Social behaviour	Particle-based	Fast, with fewer parameters to tune	Risk of premature convergence
Simulated Annealing	Metallurgy	Stochastic random walk	Effective for complex multi-modal spaces	May get stuck in local optima

In this study, GA was implemented in R using a custom script developed to optimise the selection of individuals based on the above-defined fitness function. Each candidate solution comprised exactly 50 selected individuals, enforcing a strict cardinality constraint. The initial population was generated randomly, with each chromosome encoding a feasible solution of length n and exactly 50 “1”’s. Multiple configurations were tested to explore the impact of parameter tuning. Population sizes of 100 and 250 were evaluated, with generation limits set at 100, 200, and 250. Tournament selection was employed, with tournament sizes ranging from 3 to 5 evaluated empirically. Elitism was incorporated by retaining the top 10% of individuals in each generation to preserve the best solutions. A one-point random crossover mechanism was used to combine parent chromosomes, with crossover rates tested between 0.6 and 0.9 to balance exploration and exploitation of the search space. To introduce diversity and prevent premature convergence, a swap mutation was applied by randomly flipping selected positions in the selection vector from “0” to “1” or vice versa, while ensuring the total number of selected individuals remained constant. Mutation rates between 0.05 and 0.2 were tested. To mitigate stagnation, a re-initialisation mechanism was triggered if no improvement in best fitness was observed over three consecutive generations. In such cases, 50% of the population was replaced with new, random, feasible solutions.

#### Differential evolution (DE)

DE is an evolutionary algorithm inspired by the principles of natural selection, similar to GA, but it operates on real-valued vectors rather than binary representations (Table 1), and new candidate solutions are generated through a combination of mutation and crossover operations. A key feature of DE is its differential mutation, where the scaled difference between randomly chosen vectors guides the mutation process. This differs from GA, where crossover plays a complementary role. The initial population was randomly generated within predefined bounds, with each solution representing 50 selected individuals. Population size was fixed at 100 or 200 to ensure diversity at the start of the optimisation. If the trial vector exhibited better than the corresponding target vector, it replaced the current individual in the next generation.

An adaptive selection strategy was employed in this study, where one of three standard strategies—*rand/1/bin*, *best/1/bin*, or *current-to***-***best/1/bin*—was randomly selected for each individual. The “1” in these strategies indicates the use of one difference vector in the mutation process, and “bin” refers to binary crossover, where the trial vector is formed by combining elements of the mutant and target vectors based on a crossover probability. The *rand/1/bin* involves selecting three random individuals and adding the weighted difference of two to the third. best/1/bin uses the best-performing individual as the base vector, adding the weighted difference between two others, and *current-to-best/1/bin* combines information from the current and best individuals with additional differential guidance from random vectors.

Two critical parameters influence the DE’s behaviour: the scaling factor (F) and the crossover probability (CR). The scaling factor F (tested in the range of 0.6 to 0.8) controls the magnitude of the mutation step and was adaptively varied by ± 10% to enhance population diversity and reduce the risk of premature convergence. Similarly, the crossover probability CR (range of 0.6–0.9) dictates the likelihood of mixing genes from the mutant and parent vectors and was also adaptively perturbed to balance exploration and exploitation dynamically throughout the search.

#### Particle swarm optimisation (PSO)

PSO is a population-based metaheuristic inspired by the social behaviour of swarms, where particles represent potential solutions that navigate the search space by adjusting their velocities and positions (Table 1). Although PSO was originally developed for continuous and unconstrained problems, adaptations were necessary to address the discrete and constrained nature of the problem in this study.

Each particle encoded a solution that selected exactly 50 individuals to ensure feasibility. After each velocity and position update (Elsayed et al. 2013; Parsopoulos and Vrahatis 2002), a constraint-handling mechanism was applied to correct violations by adding and removing individuals as needed. This ensured all particles remained within the feasible region throughout the optimisation process. These particles update their velocities and positions at each iteration, using a combination of their own experience (personal best), the experience of neighbouring particles, and the global best found by the entire swarm. The standard PSO velocity update equation was used in this study (Eq. 2).

2 $$v_{ij} \left( {t + 1} \right) \, = \, wv_{ij} \left( t \right) \, + \, c_{1} r_{1} \left( {p_{ij} \left( t \right) - x_{ij} \left( t \right)} \right) \, + \, c_{2} r_{2} \left( {g_{j} \left( t \right) - x_{ij} \left( t \right)} \right)$$

Here, *v*_*ij*_*(t)* represents the velocity of particle *i* in dimension *j* at iteration *t*, and *x*_*ij*_*(t)* is the current position. The personal best position is *p*_*ij*_*(t),* and *g*_*j*_*(t)* is the global best position. The cognitive component *c*_*1*_, the social component *c*_*2*_, and random variables *r*_*1*_ and *r*_*2*_ introduce stochasticity to the search process. A range of values for the cognitive and social components were tested, with *c*_*1*_ = 1.5 and *c*_*2*_ = 2 chosen for the final configuration. The inertia weight *w*, which controls the trade-off between exploration and exploitation, was set to decrease linearly from 0.7 to 0.6 across generations, promoting more exploitation as the algorithm converges. A global topology was used, allowing each particle to access the best-performing solution across the entire swarm. This ensured that information from the global best was shared across the swarm, facilitating convergence towards optimal solutions.

The initial population was randomly generated to satisfy the selection constraint (where exactly 50 individuals per solution). Throughout the optimisation, feasibility was strictly enforced. While particles could temporarily move into infeasible regions during position updates, these solutions were corrected before evaluation and were excluded from personal or global best updates. This guarantees that all solutions remain valid throughout the optimisation process. Although this method ensures feasible solutions, one drawback is that random initialisation might be time-consuming if the feasible space is limited. Additionally, it restricts exploration in infeasible regions, which might contain potential pathways to better solutions.

#### Simulated annealing (SA)

SA is an optimisation algorithm inspired by the thermodynamic process of cooling materials to form crystalline structures (Table 1). Unlike population-based algorithms such as GA, DE, and PSO, which evolve multiple solutions simultaneously, SA iteratively improves a single solution over time. The implementation commenced by generating a random initial solution, ensuring exactly 50 individuals were selected to meet the required selection constraints. At each iteration, a neighbouring solution is generated by making a small modification to the current solution, such as flipping the selection status of one individual. The *Metropolis* criterion was employed to decide whether to accept a new solution. If the new solution had a higher fitness, it was accepted outright. Otherwise, it may still be accepted with a probability that decreases as the solution quality worsens and as the temperature parameter cools. This probabilistic acceptance mechanism allows the algorithm to escape local optima and promotes exploration of the search space.

The temperature parameter, which controls the probability of accepting worse solutions, was gradually decreased following a predefined cooling rate. Early in the search, a higher temperature enables greater exploration; as the temperature decreases, the algorithm shifts towards exploitation, focusing on refining the best solutions. Various initial temperature and cooling rate combinations were tested. Finally, the initial temperatures were set to 0.01, and cooling rates were varied around 0.96 to assess their effect on convergence and solution quality. Since SA operates on a single solution at a time, its sequential nature makes parallel evaluation less applicable compared to population-based algorithms, where multiple solutions can be evaluated simultaneously.

During optimisation, fitness metrics were recorded across all generations to evaluate algorithm performance and convergence behaviour. Specifically, the average fitness of the population at each generation was tracked to monitor overall search progress, while the best fitness value represented the top-performing individuals at each stage. A fixed random seed was applied to all runs to ensure reproducibility. The final solution for each algorithm was selected as the individual with the highest fitness value in the last generation, representing the most optimal candidate under the given fitness function. Convergence speed was also considered, reflecting the number of iterations or the time required for each algorithm to reach its best fitness value. Finally, computational efficiency was measured by examining the time taken and the computational resources required by each algorithm, providing insights into the trade-offs between solution quality and resource utilisation.

A consistent experimental framework was used to ensure a fair comparison across all metaheuristic search algorithms (MSAs). Identical population sizes (100 and 200) and generation counts (100 and 250) were applied uniformly to GA, DE, and PSO. The number of iterations for SA was scaled to provide a comparable computational workload. Custom R scripts were developed for each algorithm to allow tailored implementation and parameter control. All experiments were executed on high-performance computing (HPC) infrastructure provided by the University of Queensland. The R-codes are made available on GitHub at https://github.com/SimmiSudhir/Linkage-Disequilibrium-Algorithm.git.

### Haplotype analysis of OzWheat panel

The OzWheat panel comprises 583 wheat cultivars and landraces representing diversity in global and Australian Wheat accessions (Hyles et al. 2024). Stripe rust field infection trials were conducted in 2023 across three locations in Australia. For this study, the Horsham trial(AgVic) dataset was used due to its high variation in disease scores and broad coverage, which is essential for effectively comparing the performance of different MSAs. Infection scores were recorded on a scale from 1 to 9, with 9 indicating high susceptibility. All lines were genotyped using 29,972 highly polymorphic markers filtered from the 90 K SNP array (Wang et al. 2014). Quality filtering included minor allele frequency, missingness, and marker information across the panel.

The best linear unbiased estimates (BLUEs) were calculated for the stripe rust scores using a linear model with genotypes treated as fixed effects suitable for downstream genomic analyses. Trial reliability was quantified as broad-sense heritability, calculated from a linear mixed model with genotypes considered random effects. The heritability estimate was derived from the ratio of genetic variance to total phenotypic variance, using phenotypic variance components fitted via the ASReml-R package (Butler et al. 2009). The trial employed a simple randomised plot design with two replicates per genotype.

To define haplotype blocks and calculate haplotype effects within each block, we followed the approach of Voss-Fels et al. (2019). First, SNP effects were estimated using ridge regression-best linear unbiased prediction (RR-BLUP) with the *SelectionTools* R package. The statistical model used for this analysis was:

3 $$Y = X\beta + Zu + e$$

where Y is the vector of phenotypic observations (stripe rust infection scores), X and Z are design matrices, *β* is the vector of fixed effects, *u* is the vector of random SNP effects, and *e* is the residual error.

Haplotype blocks were constructed using a linkage disequilibrium (LD)-based approach, implemented via the *SelectionTools* version 21.3 package in R. For each chromosome, pairwise *r*^2^ values were calculated between SNP markers to measure the strength of LD, indicating how strongly SNP pairs are inherited together. SNPs were grouped into blocks when adjacent markers had *r*^2^ values exceeding 0.5 (Tong et al. 2024). Flanking markers were included if their *r*^2^ values with the block’s boundary markers also met the threshold. Each block represented a haplotype composed of tightly linked SNPs inherited together with minimal recombination. Each individual in the population carried two haplotypes for any chromosomal region. SNPs not meeting LD thresholds were treated as single-marker blocks. Haplotype effects were estimated by summing the additive effects of SNPs within each defined haplotype block as described by the model in Eq. (3).

### Simulation experiment to evaluate long-term genetic gain and genetic diversity

**Fig. 2:**
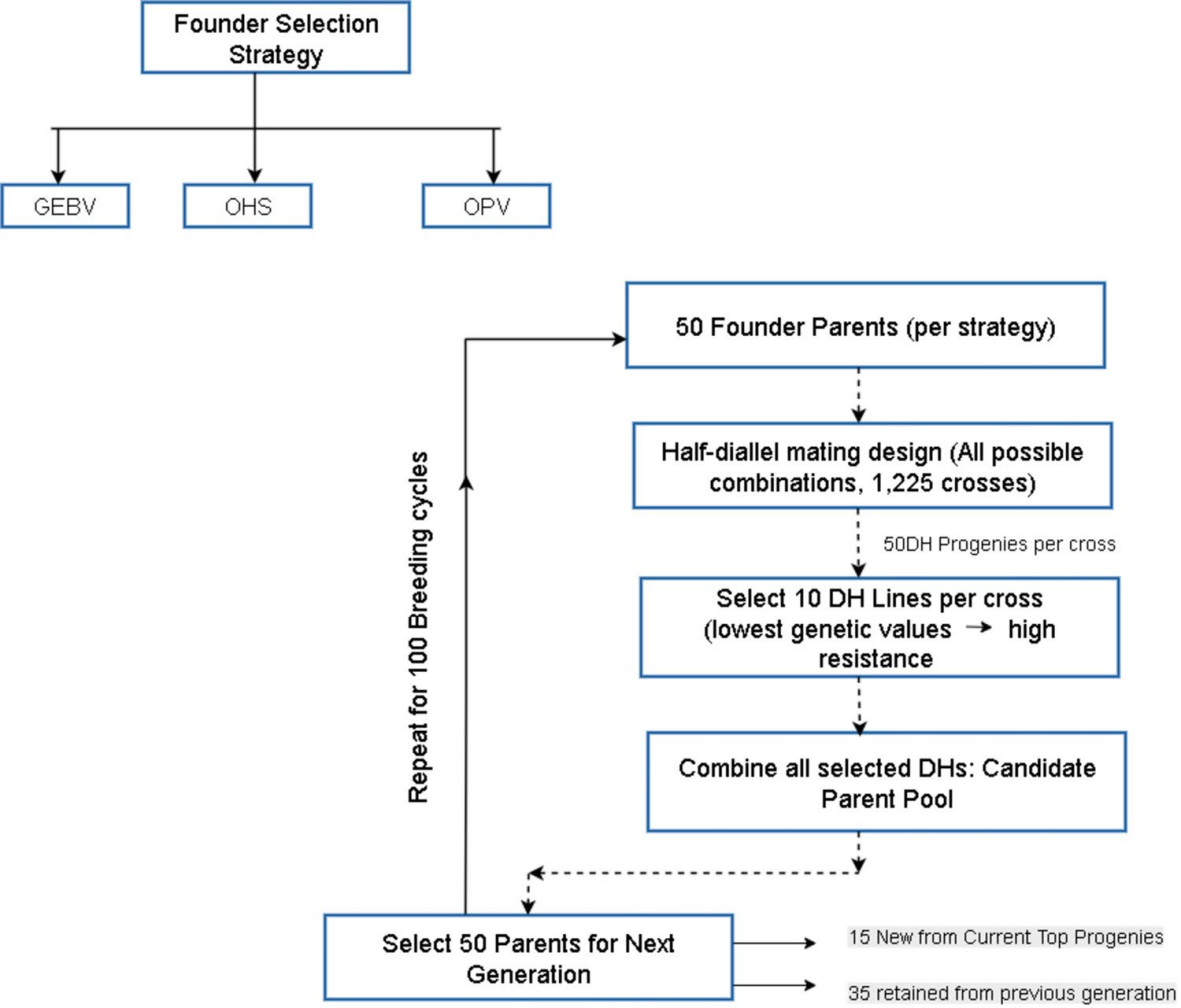
Schematic overview of the recurrent breeding simulation structure used to compare founder selection strategies

The breeding simulation was conducted using the AlphaSimR (Gaynor et al. 2021) package to evaluate the long-term impact of different founder selection strategies on genetic gain and diversity. The simulation began with founder populations selected using three strategies: (i) OHS, (ii) OPV, and (iii) GEBV-based truncation selection (Fig. 2). Each strategy was applied to the same real marker dataset comprising 29,972 SNPs and the physical map of the wheat genome. A half-diallel mating design was employed, generating all possible crosses (1225) between the selected parents. For each cross, 50 double haploid (DH) progenies were simulated, and from these, 10 DH lines per cross were selected based on their breeding values to move forward as parental lines in the next cycle. This selection targeted individuals with the lowest genetic values, representing increased resistance. These selected DH lines then served as the parents for subsequent breeding cycles (Fig. 2).

To reflect practical constraints in breeding programmes, fifteen new parental lines were selected from the highest-performing progenies of the current generation. The remaining thirty-five parents were retained from the previous generation to maintain genetic continuity (Fig. 2). This approach balances genetic gain with the preservation of background diversity, limiting turnover in a way that mirrors realistic breeding strategies. The simulation was run for 100 recurrent breeding cycles under this closed-loop structure, with no external introductions after the initial founder selection. The simulation included ten independent replicates. Across cycles, genetic gain (mean genetic value) and genetic diversity (measured by genetic variance among selected parents in each generation) were recorded to evaluate the long-term effectiveness of each selection strategy.

The R code for this simulation was adapted from the *GitHub* repository (Bancic et al. 2024) associated with the paper “*Plant breeding simulations with AlphaSimR*,” but the real rust data as described above were used instead of simulated data to assess better genetic gain and variance over a hundred cycles of genomic selection. This approach enabled a comprehensive comparison of the founder selection methods in terms of their effectiveness in improving stripe rust resistance while maintaining genetic diversity in the breeding population.

## Results

### Haplotype block construction

A total of 7465 genome-wide LD blocks (r^2^ > = 0.5) were constructed from 29,972 markers across all chromosomes. The number of markers per block and average block size varied between regions, chromosomes and subgenomes.

### Parameter optimisation and algorithm performance

**Fig. 3:**
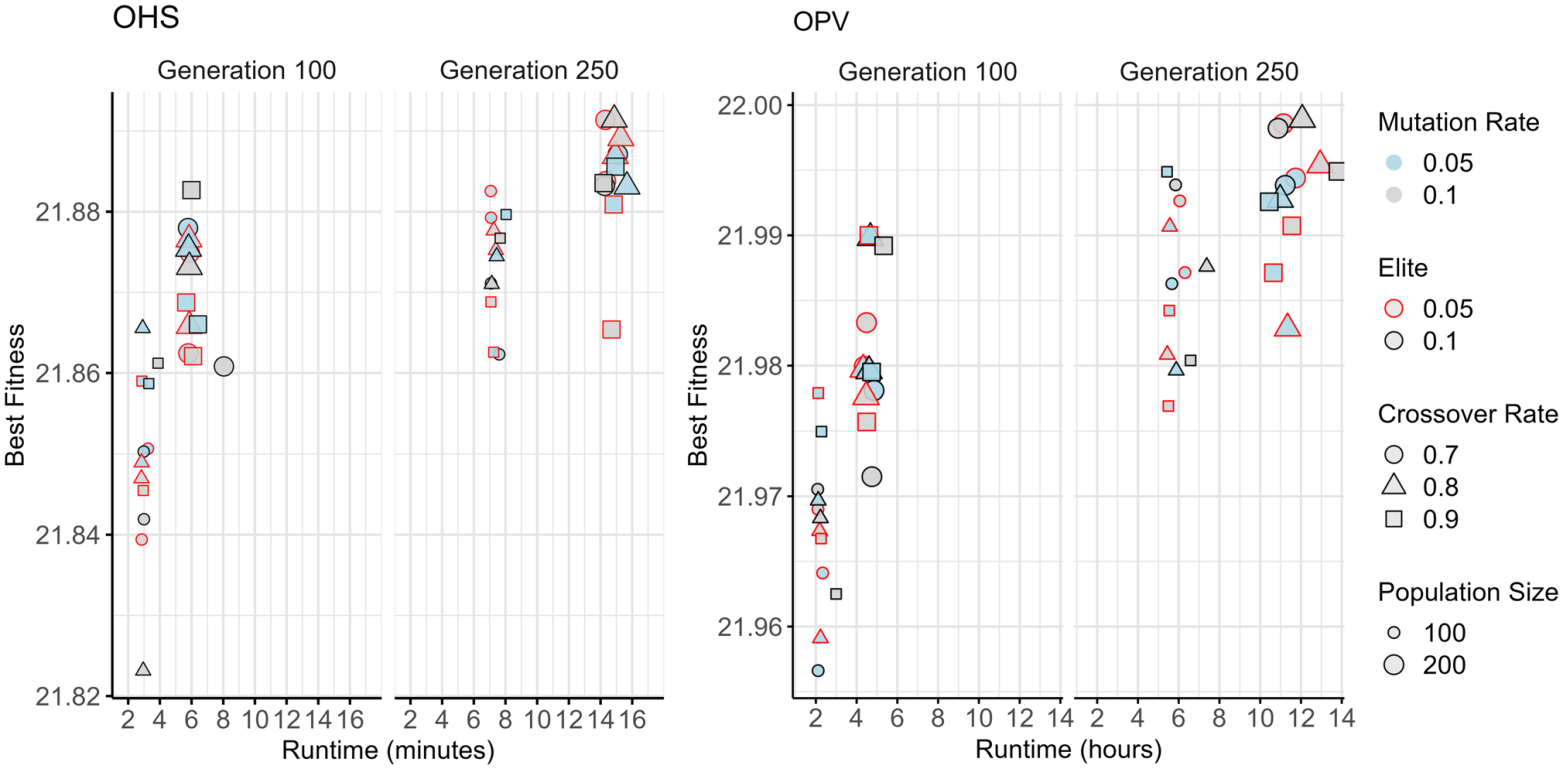
Performance of genetic algorithm (GA) on OHS and OPV fitness functions: The plots compare the best fitness achieved versus runtime for two fitness functions, OHS (left) and OPV (right), at two stages evaluated at Generation 100 and Generation 250. Each point represents a different GA configuration, varying in mutation rate (colour), elitism rate (shape border), crossover rate (shape), and population size (size). The results illustrate the trade-off between runtime and solution quality under various GA parameter settings. OHS = Optimal Haplotype Selection; OPV = Optimal Population Value

**Fig. 4:**
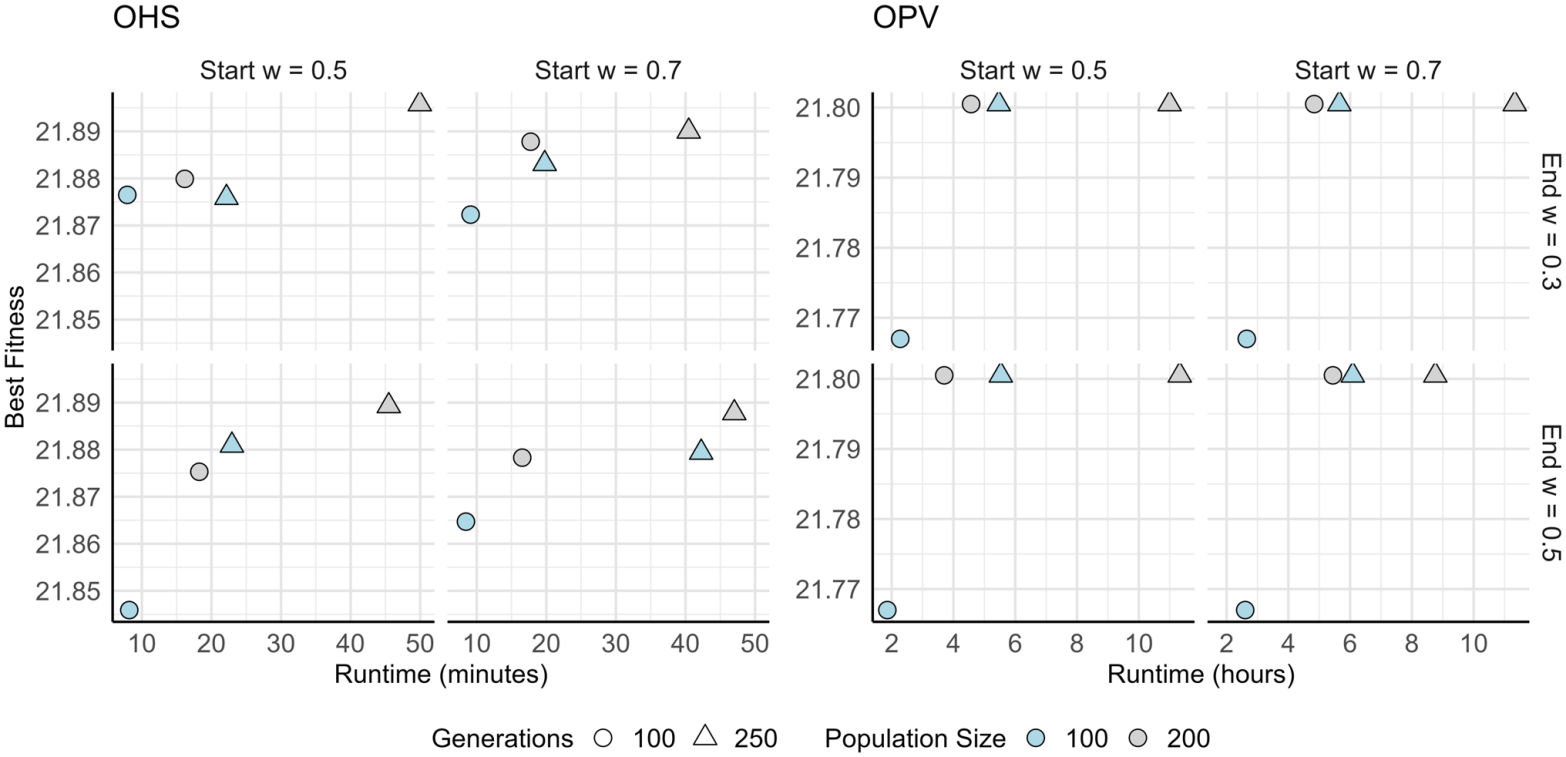
Best fitness achieved by particle swarm optimisation (PSO) on OHS and OPV fitness functions under varying inertia weights: Best fitness values are plotted against runtime for two optimisation problems: OHS (left) and OPV (right). Inertia weight schedules include starting weights w = 0.5 and w = 0.7 and ending weights w = 0.3 and w = 0.5. Fixed-weight cases (where start = end) maintain a consistent balance between exploration and exploitation. Results are reported for Generation 100 (circles) and Generation 250 (triangles), with population sizes of 100 (blue) and 200 (grey). The PSO parameters were held constant with a standard value of *c*_*1*_ (cognitive component) = 1.5 and *c*_*2*_(social component) = 2.0. The results reflect the impact of inertia scheduling and population size on convergence performance and computational cost

The key parameters for each MSA were systematically fine-tuned using a structured manual grid search approach, informed by preliminary experiments and exploratory runs. Parameters were varied systematically within empirically determined ranges, evaluating performance based on fitness optimisation, convergence speed, and consistency across multiple replicates (Table 2). For population-based algorithms (GA, PSO, DE), multiple configurations involving population size, number of generations, and method-specific parameters were evaluated across both fitness functions (OHS and OPV), visualised in Figs. 3, 4, and 6. This approach enabled the identification of robust parameter settings that generalised well across fitness functions while also highlighting trade-offs between exploration, exploitation, and computational efficiency.

**Table 2:** Key parameters for the four metaheuristic algorithms—genetic algorithm (GA), differential evolution (DE), particle swarm optimisation (PSO), and simulated annealing (SA)**—**used in this study. The table outlines the range of values tested during parameter tuning, their impact on the algorithm’s performance, and the final values selected for optimal performance across the experiments

Algorithm	Parameter	Description	Tuning consideration	Final parameters used in this study
Genetic Algorithm (GA)	Population Size	Number of Individuals in the population	Larger populations increase diversity but slow convergence; start with moderate size (e.g. 50–200) and tune as needed	200
	Crossover Rate	Probability of crossover between selected individuals	Typically set between 0.6 and 0.9. Higher values promote exploration; lower values promote exploitation	0.8
	Mutation Rate	Probability of mutation for an individual in the population	Generally small (e.g. 0.01 to 0.1), Higher values introduce more diversity but may hinder convergence	0.1
	Selection Method	Method used to select individuals for reproduction (e.g. tournament, roulette wheel)	Tournament selection increases selection pressure: the roulette wheel method is more exploratory. Tune based on population diversity	Tournament selection with size 3
	Elitism	Proportion of best individuals retained for the next generation	Set between 0.05 and 0.2. Too much elitism may lead to premature convergence	0.1
	Number of Generations	Number of iterations or generations to run the algorithm	Adjust based on computational resources and required precision. More generations allow better refinement of solutions	250
Differential Evolution (DE)	Population Size	Number of candidate solutions in the population	Larger populations promote exploration but increase computational cost. Start with a moderate size (e.g. 50–200) and adjust based on performance	200
	Mutation Factor	Controls the amplification of the difference vector. Typically F ∈ [0.4,1.0]	Higher F values promote more exploration. A lower F (e.g. 0.5) balances exploration and exploitation	0.4
	Crossover Rate	Probability of recombination between the target vector and the mutant vector (range 0 to 1)	Higher CR (e.g. 0.6–0.9) allows more exploration by combining different individuals, while lower values promote exploitation	0.8
	Strategy Selection	Mutation strategy (e.g. rand/1/bin, best/1/bin)	Choose a strategy that fits the problem. Strategies like rand/1/bin promote exploration, while best/1/bin tends to converge faster with less exploration	Adaptive strategy
	Selection Method	Greedy selection	Chooses between trial and target vectors based on fitness; replaces target if trial is superior or equal	Greedy selection (trial vs target vector)
	Number of Generations	Maximum number of generations (iterations)	More generations refine the solution but increase computational time. Adjust based on convergence criteria	250
Particle Swarm Optimisation (PSO)	Population Size	Number of particles (candidate solutions) in the swarm	Larger swarm sizes improve exploration but increase computation. Start with moderate size (e.g. 30–100) and adjust based on the problem’s complexity	100
	Inertia Weight	Controls the influence of the particle’s previous velocity on the current one. Typically *w* ∈ [0.4,0.9]	Higher *w* promotes exploration, while lower www encourages convergence. *w* is often decreased over time to shift from exploration to exploitation	0.5 to 0.3
	Cognitive Coefficient *C*_*1*_	Weight for the attraction towards a particle’s own best position (self-exploration). Standard *C*_*1*_ ~ 1.5 − 2.0	Higher c_1_ increases self-exploration, while lower values reduce individual influence. Consider using adaptive tuning for better control	1.5
	Social Coefficient *C*_*2*_	Weight for the attraction towards the global best position (social-exploration). Standard *C*_*2* ~_ 1.5 − 2.0	Higher c_2_ increases convergence towards the global best position. Tune this to balance exploration (lower c_2_) and exploitation (higher c_2_​)	2
	Velocity Bounds (v_max)	Limits the maximum velocity of particles to prevent excessive movement	Setting appropriate velocity bounds prevents particles from overshooting optimal regions. Too high may result in instability; too low may stagnate the search	Not explicitly used in this study
	Number of Iterations	Maximum number of iterations (similar to generations in GA and DE)	More iterations increase the chances of convergence to the optimal solution but at the cost of more computation. Adjust based on problem requirements	100
Simulated Annealing (SA)	Initial Temperature (T₀)	Starting temperature of the algorithm	Higher T₀ increases exploration by accepting more worse solutions early in the search	0.01
	Cooling Schedule (α)	Controls the rate at which the temperature decreases. common schedules include exponential cooling: T_k+1_ = α⋅T_k_	A slower cooling rate (α close to 1) maintains higher temperature longer, allowing more exploration. A faster cooling rate (lower α) leads to early convergence	0.96
	Markov Chain Length (Iterations per Temperature)	Number of iterations spent at each temperature level	More iterations per temperature increase the chances of exploring solutions thoroughly at each temperature	10 iterations per temperature (took longer time)
	Acceptance Probability Formula (ΔE / T)	Determines how easily worse solutions are accepted based on ΔE (change in energy) and temperature (T)	Adjust problem-specific objective scaling or parameterisation to affect how ΔE influences acceptance of worse solutions	exp((neighbor_value − current_value)/temp)

**Fig. 5:**
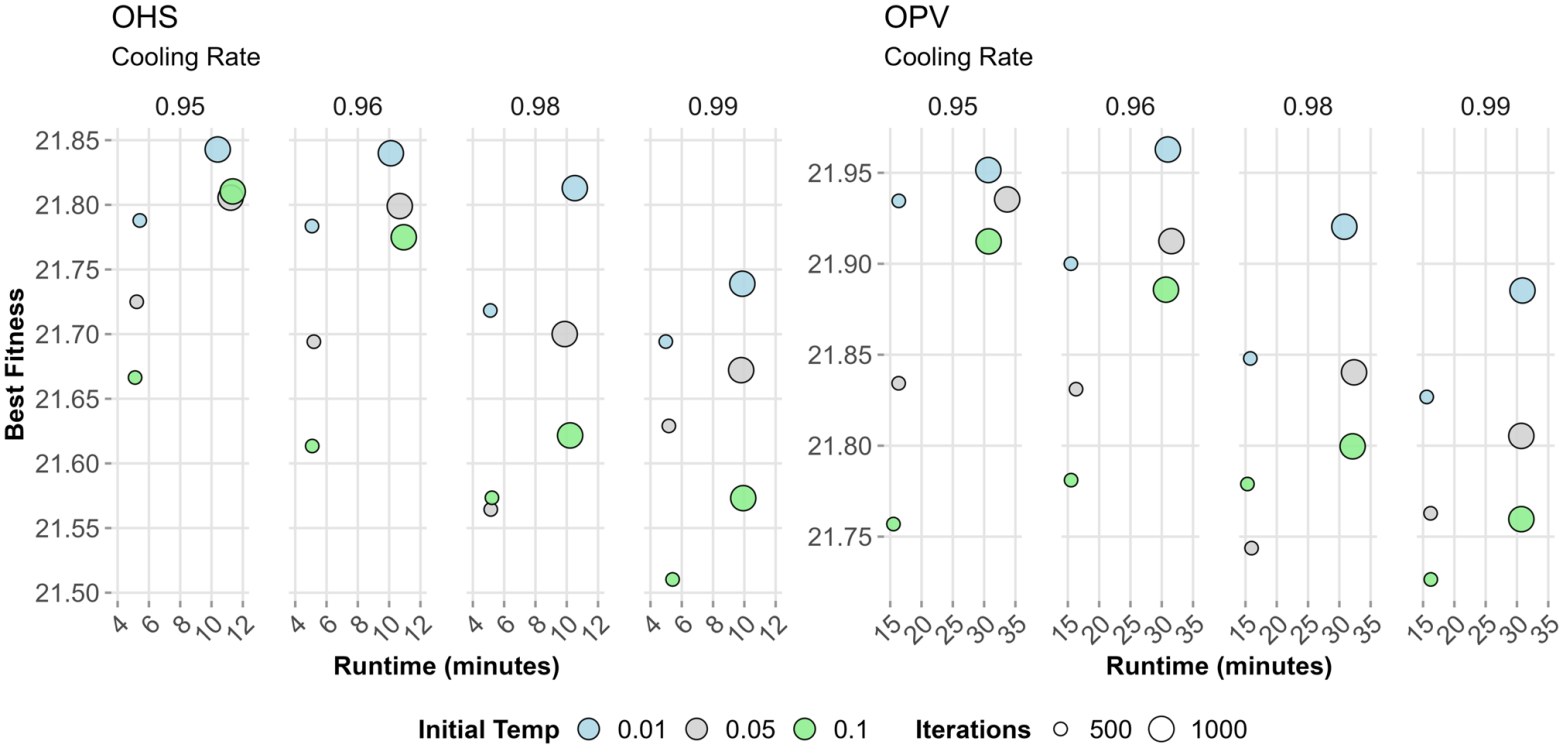
Effect of cooling rate and initial temperature settings on simulated annealing (SA) performance for OHS and OPV fitness objectives: Best fitness values are plotted against runtime for OHS (left) and OPV (right) fitness functions, evaluated across four cooling rates (0.95, 0.96, 0.98, and 0.99). Each point represents a unique SA configuration, with colour indicating the initial temperature (blue = 0.01, grey = 0.05, green = 0.1), and marker size corresponding to the number of iterations (small = 500, large = 1000). SA is a single-point search method and lacks inherent diversity, which makes it more likely to get stuck in local optima if not carefully managed with the right parameters

For GA, a range of crossover rates (0.7, 0.8 and 0.9), mutation rates (0.05 and 0.1), and elitism proportions (0.05 and 0.1) were evaluated using tournament selection (size = 2, 3 and 5). The optimal configuration comprised a crossover rate of 0.8 and a mutation rate of 0.1 with 10% elitism, as shown by stable convergence and superior fitness (Fig. 3). A stagnation detection mechanism was applied to reintroduce population diversity mid-run, effectively enhancing exploration without negatively impacting convergence speed (Table 2). PSO was assessed with fixed and dynamically decreasing inertia weights (e.g. constant at 0.5 or decreasing from 0.7 to 0.3) while keeping cognitive and social coefficients ( *c*_*1*_ = 1.5 and *c*_*2*_ = 2.0) constant (Fig. 4). For PSO, optimal performance was observed at a population size of 100 and 200 iterations for OHS; further iterations led to stagnation (Fig. 5).

**Fig. 6:**
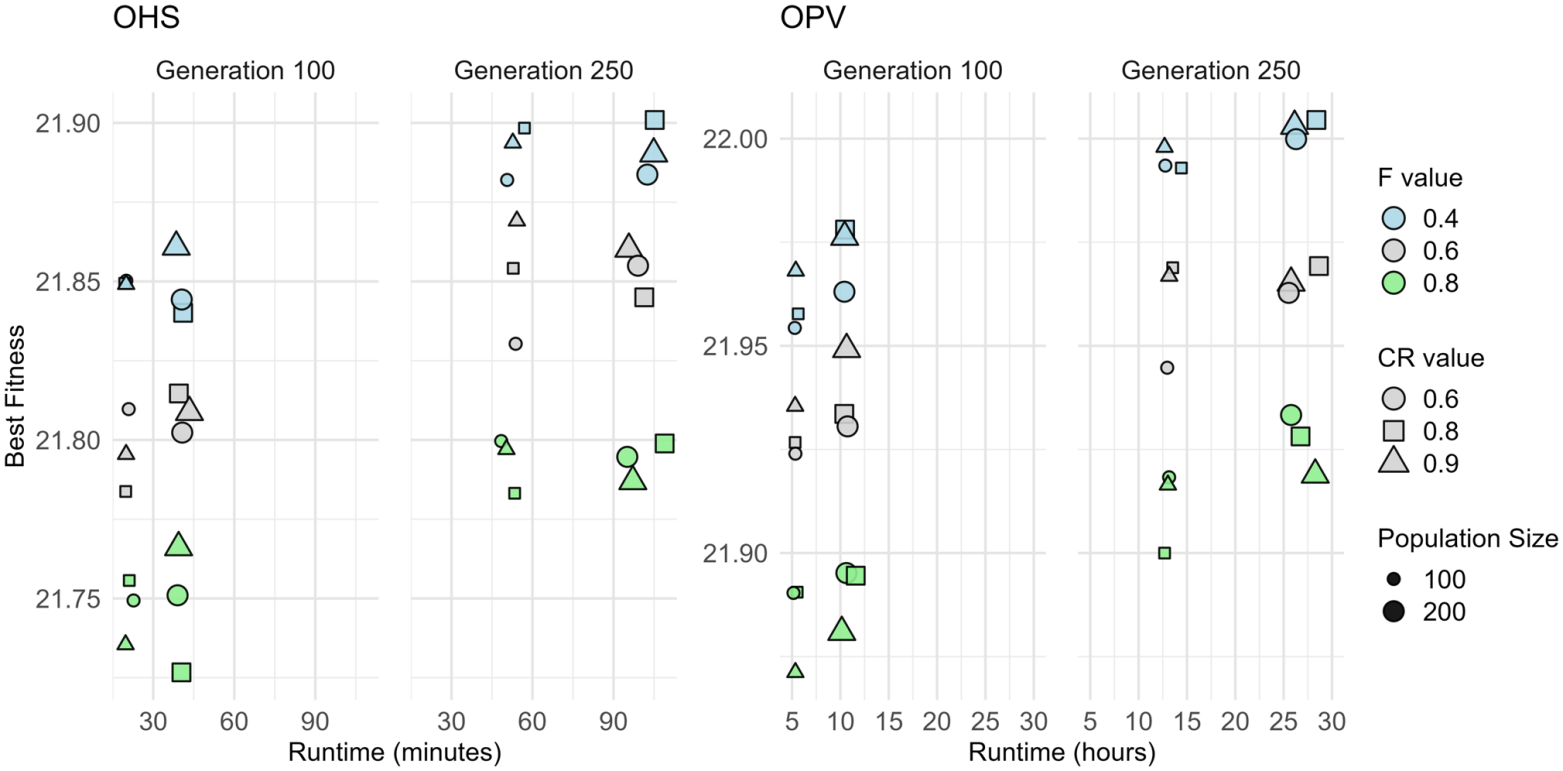
Differential evolution (DE) performance on OHS and OPV objectives across generations and parameter settings: Best fitness values and corresponding runtimes are presented for the OHS (left) and OPV (right) fitness functions at Generations 100 and 250. Each point represents a unique combination of DE parameters: mutation factor F (blue = 0.4, grey = 0.6, green = 0.8), crossover rate CR (circle = 0.6, square = 0.8, triangle = 0.9), and population size (small = 100, large = 200)

For DE, combinations of mutation factor *F* = (0.4, 0.6, 0.8) and crossover rate *CR* = (0.6, 0.8, 0.9) were tested (Fig. 6) with an adaptive selection strategy selecting between rand/1/bin, best/1/bin, or current-to-best/1/bin strategies randomly (Table 2). The configuration *F* = 0.4 and *CR* = 0.8 provided a good balance between convergence speed and fitness values for both GB metrics. DE required more iterations than GA to achieve similar fitness, reflecting its greater exploration capability and computational demand. SA was evaluated through a factorial design exploring initial temperatures (0.01, 0.05, 0.1) and cooling rates (0.95 to 0.99) (Fig. 5). Two cooling strategies, one with a Markov chain (10 iterations per temp (max temp 50 and 100)) and another with a single iteration per step, were compared (Table 2). Markov chain configurations improved exploration but incurred significant runtime costs with proportional fitness improvements.

### Computational efficiency and convergence comparison

**Fig. 7:**
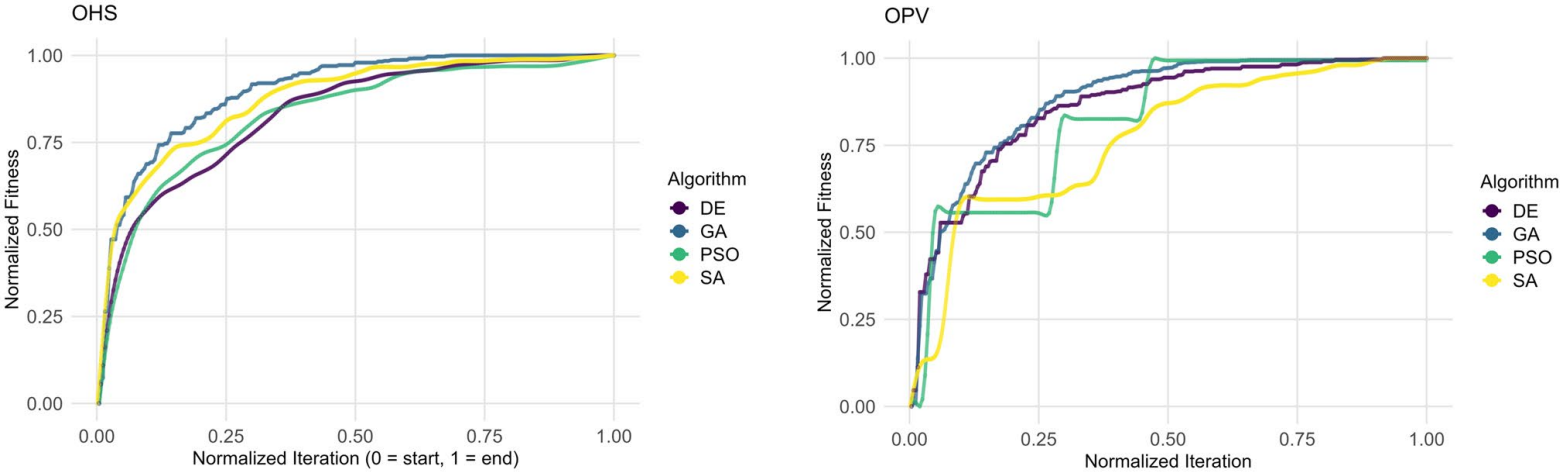
Normalised convergence curves for algorithm performance on two fitness functions, OHS (left) and OPV(right): The plots show normalised best fitness values over normalised iteration for four optimisation methods (indicated by line colour). Both fitness values and iterations are scaled to [0,1] to enable fair comparison across algorithms. Each curve represents the convergence behaviour of an algorithm, with faster and steeper ascent indicating quicker convergence towards the optimum

Computational experiments were conducted using the University of Queensland’s Bunya HPC system, with each run executed serially on a single core of AMD EPYC 7313 processors (32-core, 3.0 GHz). Algorithms were evaluated using normalised fitness versus normalised iterations to facilitate fair comparison (Fig. 7). Fitness values and iteration counts were min–max normalised within each algorithm, allowing unbiased visualisation of convergence behaviour. GA exhibited rapid, smooth convergence with a population size of 200 and 250 generations, consistently outperforming other methods in convergence speed and stability. DE demonstrated slower but consistent improvements, which were particularly effective in later optimisation stages, although at a greater computational cost. PSO, optimal at population sizes 100 and 200 iterations, experienced delayed convergence and stagnation, particularly pronounced with OPV. SA eventually achieved competitive fitness in OHS after significant iterations, but for OPV, its convergence speed was inferior to that of population-based methods, demonstrating sensitivity to cooling parameters and step sizes (Fig. 7).

OPV optimisation was generally more computationally demanding compared to OHS, as reflected in longer runtimes and delayed convergence for all population-based algorithms (Figs. 3, 4, 6). PSO particularly struggled with OPV, likely due to the complexity of the fitness landscape and inadequate swarm diversity. SA’s performance variability under OPV indicated sensitivity to cooling rates, and step size strategies (Fig. 7). However, the SA algorithm converged faster in the OPV scenario despite its inherent complexity, likely due to SA’s single-solution search approach, which reduces per-iteration computational overhead compared to population-based algorithms (Fig. 5). Notably, this supports the broader observation that, in certain cases, particularly for combinatorial optimisation problems such as the Travelling Salesman Problem, SA can exhibit faster convergence than algorithms like GA (Adewole et al. 2012). Quantitative convergence efficiency, assessed through the area under the curve (AUC), confirmed visual observations for OPV (Fig. 7). GA achieved the highest AUC (0.879), indicating superior convergence efficiency. DE closely followed (AUC = 0.860), showcasing effective refinement capabilities. PSO (AUC = 0.820) demonstrated intermediate performance with noted stagnation, while SA had the lowest AUC (0.769), reflecting its conservative exploration strategy and slower optimisation rate.

### Comparison of selected founder parents across fitness functions and algorithms

**Fig. 8:**
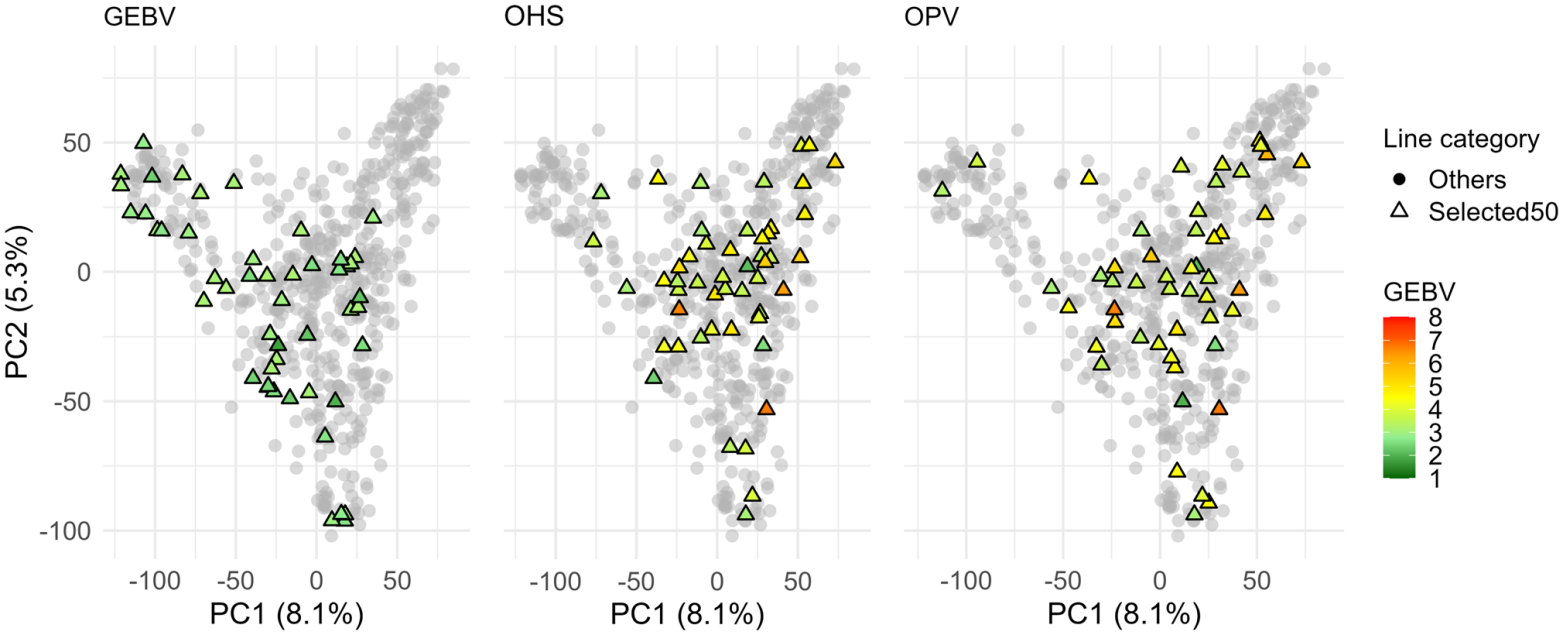
Principal component analysis (PCA) of candidate lines highlighting individuals selected under different fitness criterion: truncation based on GEBV (left) and optimisation using a genetic algorithm (GA) for OHS (centre) and OPV (right) fitness functions. Grey circles represent the full candidate set, while coloured triangles indicate the top 50 selected individuals. For all panels, colouring reflects GEBV values from low (resistant lines) to high (susceptible lines). GA was finally used for optimisation when selection is based on OHS and OPV, where it achieved superior performance in terms of maximised fitness and convergence efficiency. GEBV selection was performed via standard truncation on the top-ranking individuals

We examined the overlap among genotypes selected using three fitness functions: GEBV truncation, OHS, and OPV. The primary objective for the GB metric (OHS and OPV) was to identify genotypes that effectively combined superior chromosome segments present in the current population to construct genomes of high breeding value (Fig. 8).

**Fig. 9:**
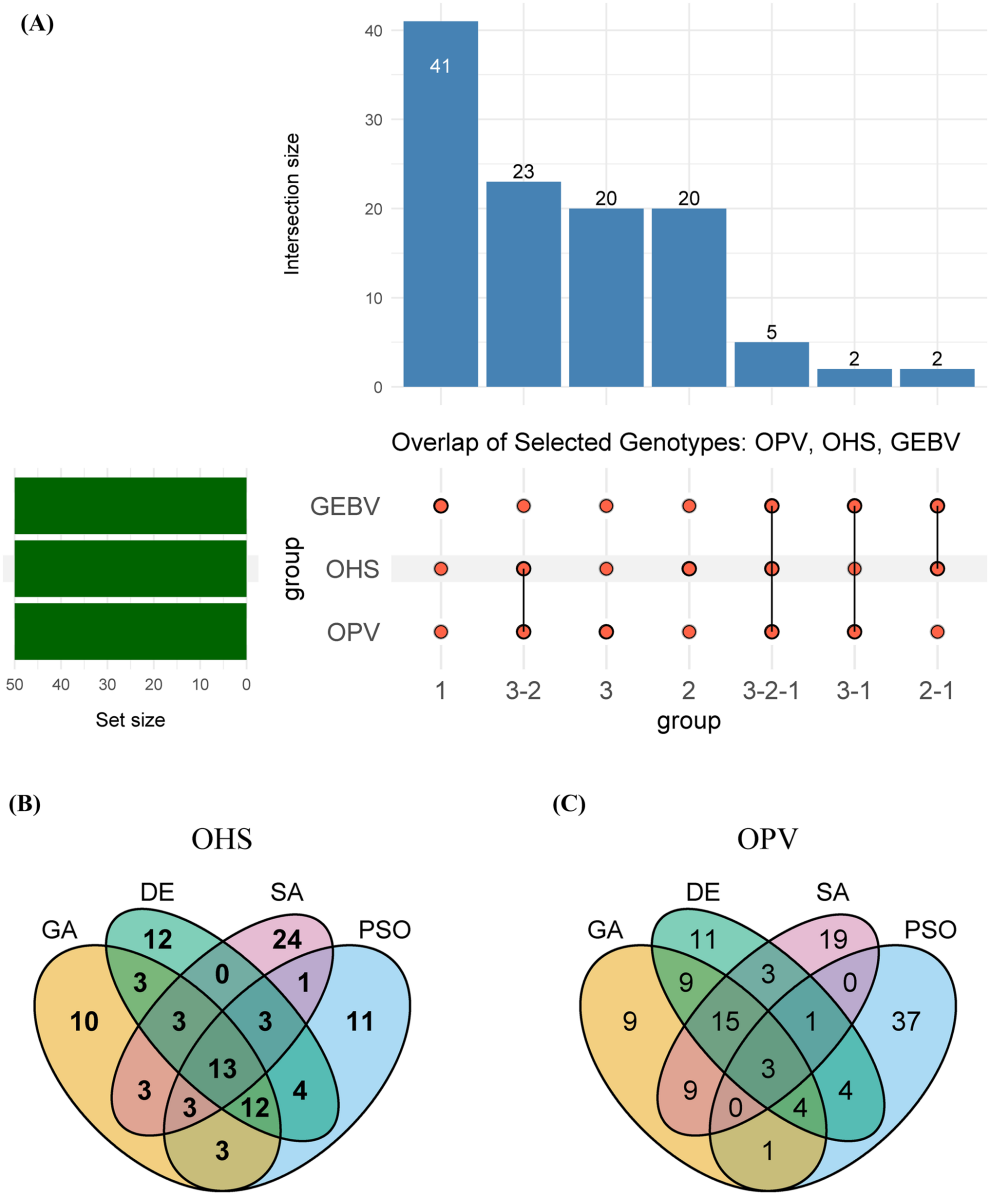
Overlap of selected genotypes across fitness functions and optimisation algorithms. **A** UpSet plot showing intersections among the top 50 selected genotypes using three fitness functions: GEBV (truncation), OHS, and OPV (optimised using GA). Vertical bars represent intersection sizes; horizontal bars show the number of selections from each method. **B** Venn diagram illustrating the overlap of selected genotypes among four optimisation algorithms (GA, DE, SA, PSO) based on the OHS fitness function. **C** Venn diagram showing the overlap of selected genotypes across the same algorithms using the OPV fitness function. Overlapping regions indicate shared selections, while unique regions reflect algorithm-specific outcomes

Notably, the genotypes selected using GEBV (comprising all resistant lines) were largely distinct from those chosen via OHS and OPV (which also included moderate to susceptible lines, Fig. 8). Specifically, GEBV-based selection shared only seven (5 + 2) genotypes, each with OHS and OPV, with just five genotypes common across all three methods (Fig. 9A). In contrast, OHS and OPV selections showed considerable overlap, sharing 28 (23 + 5) common lines (Fig. 9A). Additionally, each fitness function uniquely identified distinct genotypes, highlighting specific elements captured by each fitness criterion.

Further, we evaluated whether different optimisation algorithms selected similar parental sets within each GB fitness function. For OHS, a significant overlap of 31 genotypes was observed among parents selected by GA, DE, and PSO (Fig. 9B). Similarly, for OPV, GA and DE shared 31 parents, indicating strong concordance in parental selection (Fig. 9C).

For OPV, SA selected a relatively distinct set of parents, as evidenced by limited overlap with GA and DE (Fig. 9C), likely reflecting its unique search dynamics and sensitivity to parameter tuning. While PSO showed the highest number of unique selections (37), its poor performance in OPV limited its comparability.

### Performance of OHS, OPV, and GEBV founders across genomic selection cycles

The recurrent genomic selection simulation experiment was initiated using parental pools optimised through GA (best fitness achieved) under two fitness functions: OHS and OPV. In each selection cycle, fifteen new parents were introduced into the breeding pool, while the remaining individuals were retained from the previous generation. This design introduced controlled genetic novelty while maintaining key chromosomal regions linked to rust resistance. A standard GEBV-based selection approach was also included for comparison.

**Fig. 10:**
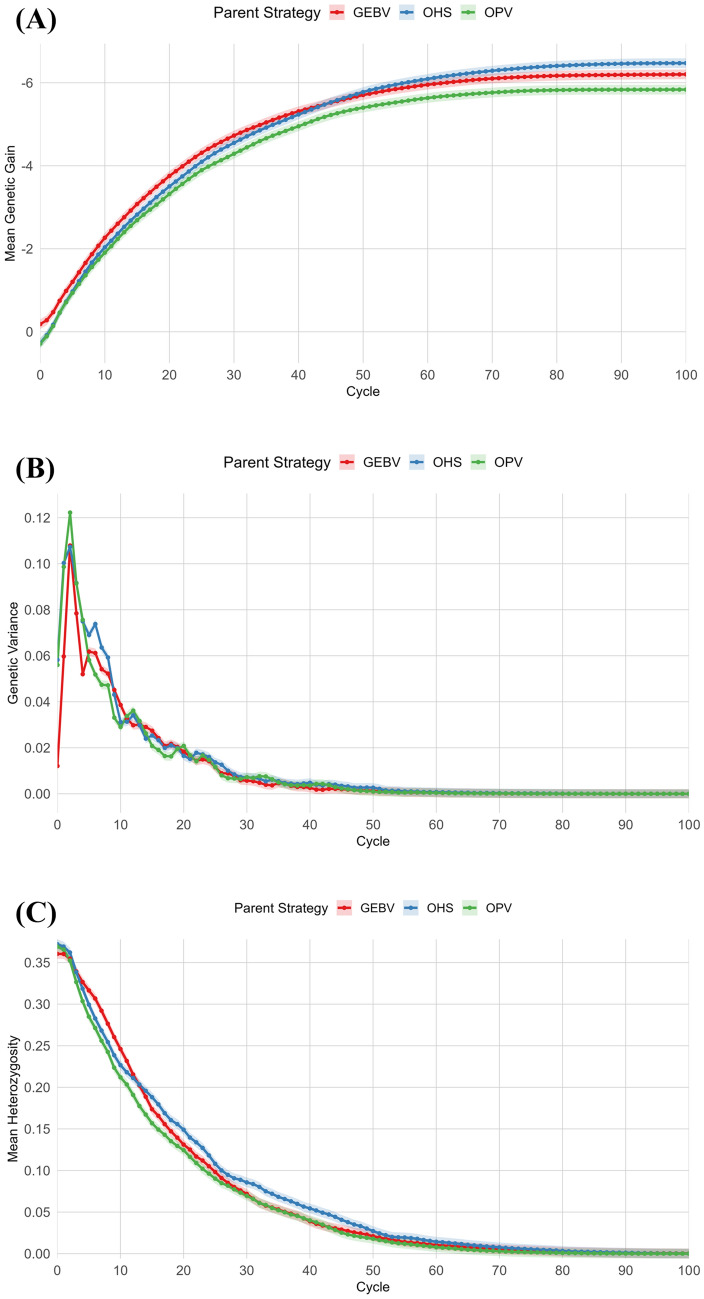
Trends in genetic gain, genetic variance, and expected heterozygosity across 100 recurrent selection cycles under three founder selection strategies: OHS, OPV, and GEBV-based truncation. **A** Cumulative genetic gain shows that OHS maintains the highest long-term gain. **B** Genetic variance declines over time for all strategies, with OHS retaining more variance in later cycles. **C** Expected heterozygosity follows a similar pattern, where OHS maintains higher levels across cycles, supporting sustained diversity compared to OPV and GEBV

OHS-based selection led to the highest cumulative genetic gain across 100 cycles (Fig. 10A). While the GEBV approach also showed strong performance**,** especially in early cycles, it plateaued sooner, possibly due to faster fixation of a narrower set of beneficial alleles, while the OPV strategy**,** which aimed to optimise long-term gain by combining the best haploid genomes, projected to produce superior progeny, underperformed in this case (Fig. 10A). Genetic variance trends (Fig. 10B) further clarify these dynamics. OPV started with the highest genetic variance, reflecting its initial diversity among selected parents. However, variance under OPV declined sharply, likely due to the truncation selection applied at the progeny level, which concentrated on individuals with high breeding values and thereby reduced diversity quickly. OHS also experienced a reduced variance but at a slower rate, indicating a better balance between selection and diversity retention. GEBV-based selection began with the lowest variance, likely because it drew from a more homogeneous set of top-scoring individuals. However, recombination over successive cycles introduced some new variation, visible as a slight increase before variance again declined as alleles approached fixation (Fig. 10B). Ultimately, variance reduced over time in all strategies due to the combined effects of directional selection and limited inflow of new alleles.

The trend in expected heterozygosity (Fig. 10C) follows a similar pattern. OHS maintained the highest heterozygosity across most cycles, indicating slower allele fixation and broader retention of polymorphisms, followed by OPV.

## Discussion

### Effectiveness of MSAs in genomic selection

This study demonstrates the robustness and efficacy of MSAs for optimising founder-parent selection under the GB framework. The inherently combinatorial nature of selecting optimal haplotype combinations presents a significant challenge and is well suited for computational intelligence approaches. By reformulating the selection problem into an objective fitness function, we effectively leveraged four widely used MSAs, including GA, DE, PSO, and SA, to two GB objectives (OHS and OPV), benchmark optimisation performance and biological outcomes in a GS context.

### Algorithm performance and computational efficiency

Overall, GA emerged as the most efficient and robust optimiser across OHS and OPV fitness functions, quickly reaching near-optimal solutions with minimum variability. This consistency underscores their reliability in handling the combinatorial nature of founder-parent selection as a group. A study by Lim and Haron (2013) also highlighted GA’s efficacy over DE and PSO across standard benchmark functions.

DE performed comparably in final fitness but took longer to run due to higher computational cost per iteration. While it is known for its robustness, it required extensive parameter tuning to optimise both fitness functions used in this study. The convergence delay in DE was primarily due to its sensitivity to step size within our case study. Despite implementing an adaptive strategy to mitigate this issue, DE’s computational demands remained higher than those of PSO and GA. This suggests that DE’s effectiveness is highly contingent on precise parameter settings, potentially limiting its practicality in scenarios with restricted computational resources or time constraints. This observation resonates with findings from Pattanaik et al. (2019), who reported significant performance dependency on problem-specific tuning for DE.

PSO achieved the best performance for the OHS objective. This aligns with its recognised efficiency in navigating complex search spaces, as outlined by Phan-Van et al. (2023) and Soltani-Mohammadi et al. (2016), and supports its suitability for breeding optimisation tasks where rapid and effective solutions are required. However, it struggled with premature stagnation and abrupt convergence steps for OPV, likely due to the more complex and rugged fitness landscape. Although effective in isolated cases, SA was generally outperformed by population-based methods and showed greater sensitivity to its cooling rate and step size configuration. While initially lagging behind the population-based algorithms in fitness optimisation, SA showed improved performance after increasing the number of iterations to 1000. However, its single-solution approach restricted the feasibility of parallel evaluations, affecting its computational efficiency compared to other algorithms. Despite these limitations, SA’s efficiency in scenarios with constrained computational resources suggests it remains a viable option, particularly when alternative stopping criteria are used instead of a fixed number of iterations to achieve convergence. This is supported by Adewole et al. (2012), who noted SA’s faster runtimes in solving the Travelling Salesman Problem despite not always matching the solution quality of GA.

Fernandes and Yen (2019) and Moayedi et al. (2019) demonstrated PSO’s superior performance in optimising artificial neural networks (ANN) and convolutional neural networks (CNN) compared to standard models. In our study, PSO also showed strong performance, particularly in terms of convergence speed and fitness, though this was more evident for the OHS metric than for OPV. This contrast highlights how algorithm performance can vary depending on the specific characteristics of the optimisation problem.

The overlap between parental lines selected by different algorithms further supports these observations. GA, DE, and PSO frequently selected similar genotypes for OHS, suggesting convergence on high-performing regions of the search space. In contrast, SA often selected different parents, reflecting its distinct optimisation path and lower fitness results. These findings indicate that population-based MSAs, particularly GA and DE, are better suited to the founder selection problem, especially when combinatorial complexity is high.

The significance of computational efficiency becomes more critical as genomic datasets grow in size and complexity. In this study, we worked with 583 lines and 7465 haploblocks across 29,972 loci, a population size that is manageable with current resources. However, as breeding programmes expand, the scalability of algorithms like GA will be essential. Their ability to converge quickly allows for more iterations within a given timeframe, which enhances both the robustness and reliability of optimisation outcomes. Efficient algorithms not only improve the selection process but also facilitate their seamless integration into broader genomic selection pipelines, including data preprocessing and post-selection analyses. This ensures that the optimisation step does not become a bottleneck as the scale of genomic data increases.

Understanding the strengths and limitations of each algorithm is crucial for selecting the most suitable approach for specific tasks. While theoretical performance is important, practical factors such as computational resources and task requirements should also be considered. For instance, GA’s faster convergence and superior fitness optimisation make it ideal for large-scale tasks where computational efficiency is critical. On the other hand, DE’s robustness and ability to explore the solution space thoroughly may be more appropriate when the quality of the solution is paramount, and sufficient computational resources are available to accommodate its slower convergence (Georgioudakis and Plevris 2020). Therefore, the choice of an optimisation algorithm should be tailored to both the complexity of the task and the researcher’s practical constraints (Kachitvichyanukul 2012). To further enhance optimisation outcomes, hybrid algorithms that combine the strengths of multiple MSAs could be explored (Chen and Shahandashti 2009; Tanha, Shirvani, Rahmani 2021). These hybrid approaches may balance speed and robustness, potentially yielding better results in a specified optimised task. Additionally, investigating the scalability of these algorithms in larger genomic datasets will be essential for their application in large-scale breeding programmes. By conducting comparative analyses and evaluating scalability, researchers can make more informed decisions about which algorithms or hybrid approaches are best suited for their specific optimisation needs.

### Implications for breeding programmes

This study demonstrates the value of GB strategies, specifically OHS and OPV, for founder-parent selection in genomic-assisted breeding programmes. Both approaches leverage haplotype-level information to improve long-term breeding outcomes. Among them, OHS consistently delivered higher cumulative genetic gain and better maintained genetic diversity compared to OPV and GEBV-based selection. The superior performance of OHS likely stems from its focus on selecting actual individual lines carrying favourable haplotype blocks, particularly those associated to rust resistance. This approach helps preserve and transmission of key chromosome segments across breeding cycles. In contrast, OPV constructs a theoretical optimal genome by combining desired haploid segments, but these combinations must be realised through recombination events in the actual breeding population. Although recombination frequency was consistent across selection methods, OPV’s strategy led to fragmentation of beneficial haplotype segments when theoretical optimal contributions were not fully realisable in practice. The subsequent GEBV-based selection applied to progenies from OPV-designed founders likely accelerated the loss of these fragmented or partially assembled haplotypes, as evidenced by the sharp decline in genetic variance shown in Fig. 10B. Thus, the underperformance of OPV may be attributed to recombination as well as inability to preserve intact, favourable haplotype blocks under subsequent GEBV-driven selection.

In contrast, OHS directly selects elite founders with complete functional haplotype combinations, reducing the dependence on recombination to reassemble desired genotypes. As a result, OHS maintained long-term genetic gain and moderate levels of genetic variance and heterozygosity. These outcomes are particularly important in resistance breeding, where preserving diverse haplotypes enhances durability under evolving pathogen pressures.

GEBV-based truncation selection produced rapid genetic gains in the initial breeding cycles by selecting individuals predicted to have superior genetic merit for target traits; however, this approach plateaued earlier than OHS, likely due to the fixation of a narrow set of favourable alleles. Both OPV and OHS started with higher initial variance, but OHS maintained this advantage over time, illustrating the importance of selection strategies that directly preserve favourable genomic configurations.

While this study applied a linear mixed model to estimate SNP and haplotype effects, it assumes that phenotypic values follow a normal distribution. However, stripe rust scores are ordinal in nature. Although linear models are widely used for their computational efficiency, they may not fully capture the properties of ordinal traits. Future studies could explore threshold models that are more appropriate for categorical phenotypes and may yield more accurate effect estimations for traits like disease resistance (Wu et al. 2019).

## Future directions

To capitalise on the observed patterns and enhance long-term genetic gains, it is crucial to optimise crossing strategies that leverage the available genetic diversity and favourable alleles that are present in the parent pool, particularly in the early stages of the breeding programme. This approach should aim to maximise the recombination events that can bring together advantageous allele configurations (or haplotype configurations), as elaborated by (Hayes et al. 2024; Villiers et al. 2024). Additionally, regularly introducing new genetic material is crucial to avoid narrowing the genetic base over time.

Specifically, increasing the emphasis on heterozygosity can preserve genetic diversity and sustain long-term selection response. Insights from simulation studies should continue to inform crossing strategies and pre-breeding activities. Regular refinement of selection methods and crossing strategies to balance genetic merit with diversity will support the development of a resilient and adaptable breeding population.
